# Novel Insights into Viewer-Centered Versus Stimulus-Centered Hemispatial Neglect: A Cross-Sectional Behavioral and Imaging Study of Acute Stroke

**DOI:** 10.3390/brainsci15020208

**Published:** 2025-02-17

**Authors:** Ashley Raman, Andreia V. Faria, Michael Colavito, Argye E. Hillis

**Affiliations:** 1 Department of Neurology, Johns Hopkins University School of Medicine, 600 N. Wolfe Street, Baltimore, MD 21287-0005, USA; araman10@jh.edu (A.R.); mcolavi1@jhmi.edu (M.C.); 2 Department of Radiology, Johns Hopkins University School of Medicine, 600 N. Wolfe Street, Baltimore, MD 21287-0005, USA; afaria1@jhmi.edu; 3 Department of Physical Medicine and Rehabilitation, Johns Hopkins University School of Medicine, 600 N. Wolfe Street, Baltimore, MD 21287-0005, USA; 4 Department of Cognitive Science, Johns Hopkins University, 3400 N. Charles St., Baltimore, MD 21218, USA

**Keywords:** stroke, post-stroke cognitive impairment, neuropsychological deficits, hemispatial neglect, hypoperfusion

## Abstract

**Background/Objectives:** Hemispatial neglect is common after stroke but is often evaluated only after right hemisphere (RH) stroke. We sought to determine the prevalence of two types of neglect, viewer-centered neglect (VCN) and stimulus-centered neglect (SCN), after left hemisphere (LH) and RH strokes. Additionally, we identified lesion load in each vascular territory and areas of hypoperfusion, estimated with FLAIR hyperintense vessels (FVHs) that contribute to neglect. **Methods:** A series of 233 stroke patients (73 LH and 160 RH) were administered a task to detect VCN and SCN and received brain MRI within 5 days of onset. We used multivariable logistic regression to identify vascular territories where lesion load and/or hypoperfusion contributed to each type of neglect. **Results:** While VCN was more prevalent after RH stroke, SCN occurred at a similar rate after LH and RH stroke. In RH stroke, lesion load in the middle cerebral artery occipital region and anterior cerebral artery territory and age predicted left VCN, whereas parietal hypoperfusion independently predicted left SCN. In LH stroke, lesion load across regions and age predicted right VCN, while lesion load in posterior cerebral artery occipital and anterior cerebral artery regions, as well as age, predicted right SCN. The addition of information about hypoperfusion improved the prediction of both VCN and SCN. **Conclusions:** VCN and SCN are each common after RH stroke, and SCN is common after LH stroke. Each type of neglect is accounted for by distinct areas of infarct and hypoperfusion. Results will aid in the detection of neglect after stroke and may guide reperfusion interventions to improve neglect.

## 1. Introduction

At least since the mid-1990s, it has been recognized that hemispatial neglect can occur in distinct reference frames [1,2]. In viewer-centered neglect (VCN; also called egocentric neglect), the contralesional side of the viewer or environment (what the viewer sees) is neglected. In stimulus-centered neglect (SCN; also called allocentric neglect), the contralesional side of each stimulus is neglected, irrespective of the location with respect to the viewer. Both left hemisphere (LH) and right hemisphere (RH) strokes can cause contralesional VCN or SCN [2,3,4,5,6]. However, most studies indicate that VCN is more common than SCN. Most of these studies have included only right hemisphere stroke [7,8,9]. However, two studies that have also included left hemisphere stroke have found that SCN is more common than VCN in left hemisphere stroke, while VCN is more common in right hemisphere stroke [3,10].

A few investigators have questioned the dissociation between VCN and SCN because of the common co-occurrence [11] or reported that each type of neglect can be detected in the same participants, but with different tasks or task demands [12]. However, several studies using the same task and task requirements to detect each have shown a clear dissociation or even double dissociation between VCN and SCN [4,9].

Several studies have attempted to identify the lesions associated with VCN versus SCN. Some have studied participants with chronic or subacute stroke. This approach can miss some critical lesion sites because participants with those lesions may have recovered. The studies of later strokes show areas where recovery of neglect is unlikely, whereas studies of acute strokes show areas that, when damaged, cause neglect (before the opportunity for reorganization or recovery). Nevertheless, there are some commonalities across studies, such as more frontal or dorsal lesions associated with VCN and more temporal or ventral lesions associated with SCN [5,7,13,14,15]. Studies that have evaluated the influence of age have also shown that older age is associated with greater frequency and severity of any neglect [16].

**Figure 1:**
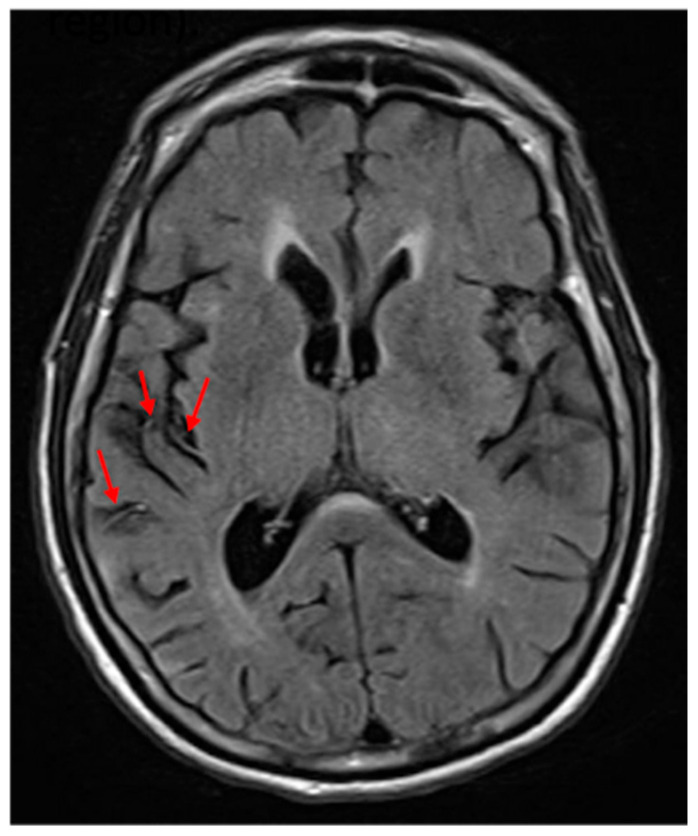
FLAIR hyperintense vessels (two in the middle cerebral artery frontal region and one in the insular region). The red arrows point to the FLAIR hyperintense vessels.

One limitation in studying individuals with acute stroke is that areas of hypoperfusion beyond the infarct may be responsible for acute deficits [17,18]. Studies using perfusion imaging have shown that in acute stroke, hypoperfusion beyond the infarct contributes to deficits, including neglect [17,19,20]. Studies have also shown that reperfusion of dorsal regions results in improvement of VCN, and reperfusion of ventral regions results in recovery of SCN [21]. However, perfusion imaging is not always available. In its absence, previous work has shown that FLAIR hyperintense vessel (FHV) number and site can be used to estimate the volume [22,23] and site [24] of hypoperfusion. An FHV (see Figure 1) indicates slow blood flow in that area, and the total number can be used to estimate the volume of hypoperfusion [22]. Furthermore, the site of the FVH is strongly associated with the site of significant hypoperfusion [24].

In this study, we tested three hypotheses in a relatively large number of individuals with acute ischemic stroke with a single task and MRI of the brain within 5 days of stroke: 1. VCN and SCN dissociate, even on the same task/with the same task demands, and VCN is more common after right hemisphere stroke, while SCN is more common after left hemisphere stroke. 2. Partially distinct areas of infarct contribute to VCN and SCN. 3. Information about the areas of hypoperfusion (estimated with FLAIR hyperintense vessels) contributes additional information to areas of dysfunction (infarct and/or hypoperfusion) that account for the variance in each type of neglect.

## 2. Materials and Methods

This study was approved by the Johns Hopkins Institutional Review Board (protocol: NA_00042097). A consecutive series of 233 individuals with acute ischemic stroke (73 LH and 160 RH) who provided informed consent were enrolled. Inclusion criteria included the following: the ability to complete testing and MRI within 5 days of ischemic stroke; the ability to provide informed consent or indicate a surrogate to provide informed consent; the ability to understand directions for the task; and premorbid competency in English by self-report. Exclusion criteria included the following: neurological disease involving the brain other than stroke; prior symptomatic stroke (asymptomatic lacunar infarcts were not excluded); uncorrected hearing or visual acuity loss; and reduced level of consciousness or ongoing sedation. Every attempt was made to enroll patients within 24 h of admission to the hospital. Patients were tested seven days per week. Fewer left hemisphere stroke survivors were included because of difficulty understanding the task in the presence of aphasia.

**Figure 2:**
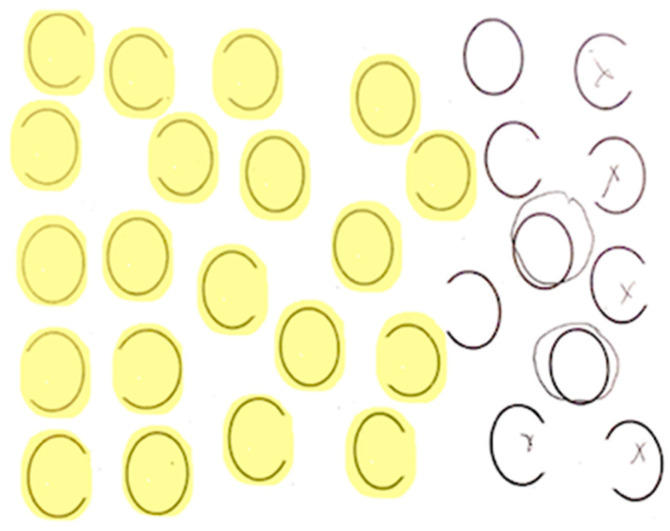
Example of left viewer-centered neglect (VCN). Participants were asked to circle full ovals and cross out (put an X on) ovals with a gap on either side. The yellow highlighted items indicate errors. Errors were stimuli that the patient failed to mark at all.

**Figure 3:**
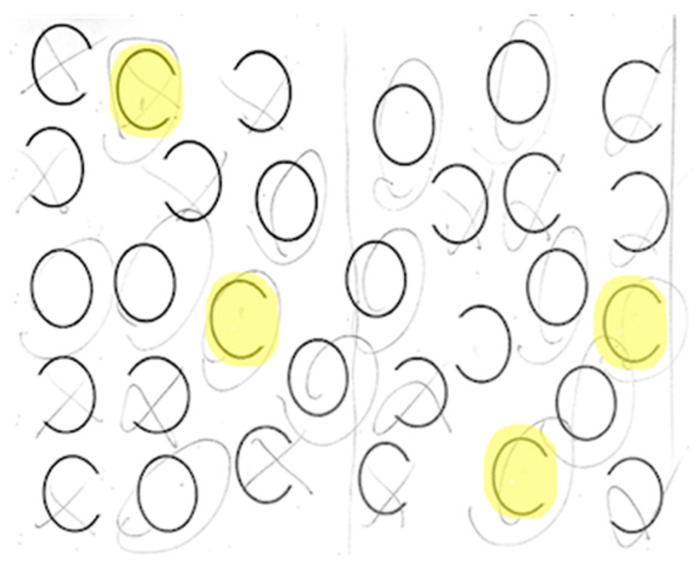
Example of right stimulus-centered neglect (SCN). Participants were asked to circle full ovals and cross out (put an X on) ovals with a gap on either side. The yellow highlighted items indicate errors. Errors were gaps (on the right) that the patient failed to notice.

Each participant completed a neglect task with 30 ovals: 10 full, 10 with a gap on the left, and 10 with a gap on the right (adapted from Ota). Participants were asked to circle full ovals and cross out ovals with a gap on either side. We defined VCN as >10% of the total number of ovals left unmarked after the most extreme mark on the contralesional side (Figure 2) and SCN as incorrectly marking >10% of contralesional gaps (Figure 3). All participants completed neglect testing and brain MRI within 5 days of stroke onset.

On FLAIR sequences, we identified FHVs in 4 MCA regions, according to a published method [22]: frontal, temporal, parietal, and insular, as well as ACA and PCA territories (Figure 1). Each region was scored from 0 to 2: 0 = no FHVs; 1 = 1–2 FHVs on 1–2 slices; 2 = 3 or more vessels on 1 slice; or 3 or more slices with at least 1 FHV (total = 0–12). The stroke core was automatically delineated with ADS (https://www.nitrc.org/projects/ads, accessed on, https://www.nature.com/articles/s43856-021-00062-8, accessed on 1 December 2024) and revised by an experienced neuroradiologist (AVF). The vascular territories affected were defined according to the digital atlas of arterial territories (https://www.nitrc.org/projects/arterialatlas, accessed on 1 December 2024, https://www.nature.com/articles/s41597-022-01923-0, accessed on 1 December 2024). Total infarct volume and lesion load in the right and left anterior cerebral artery, middle cerebral artery—frontal, middle cerebral artery—parietal, middle cerebral artery—temporal, middle cerebral artery—occipital, posterior cerebral artery—temporal, and posterior cerebral artery—occipital were calculated using the pipeline that utilized b0, Diffusion Weighted Imaging (DWI), Apparent Diffusion Coefficient (ADC), and Fluid Attenuated Inversion Recovery (FLAIR) sequences.

We used multivariable logistic regression, with the presence of VCN or SCN in each hemisphere as the dependent variables and FHV ratings in each area, lesion load in each territory, and age as independent variables.

## 3. Results

There were no significant differences between left hemisphere and right hemisphere stroke patients with respect to age, sex, racial distribution, or infarct volume. There was a marginally significant difference in education, with more years of education in the left hemisphere stroke patients (mean 14.4 versus 13.5). Right hemisphere stroke patients showed a numerically higher average volume of infarct and a significantly larger volume of hypoperfused tissue estimated by FVH. Demographics are shown in Table 1.

### 3.1. Dissociation Between VCN and SCN and the Incidence of Each After Left and Right Hemisphere Stroke

**Figure 4:**
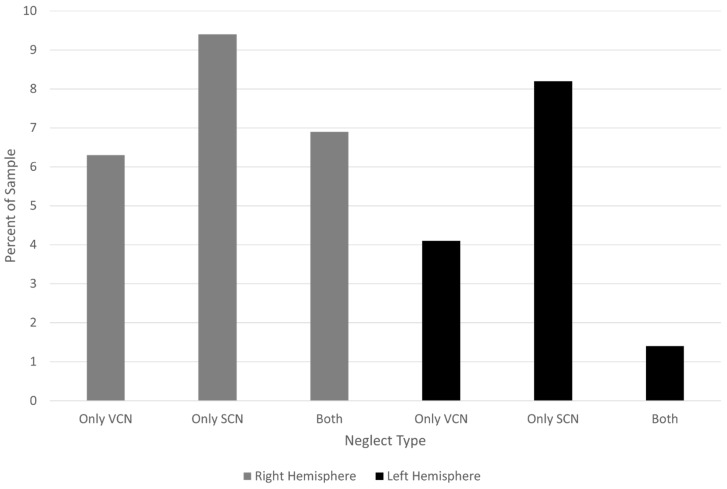
Distribution of neglect types due to strokes in each hemisphere.

Among participants with right hemisphere stroke, 21 (13.1%) had VCN (with or without SCN), 26 (16.3%) had SCN (with or without VCN), and 11 (6.9%) had both VCN and SCN. Among participants with left hemisphere stroke, four (5.5%) had VCN (with or without SCN), seven (9.6%) had SCN (with or without VCN), and one (1.4%) had both VCN and SCN. Figure 4 shows the percentage with VCN and SCN alone in each hemisphere group.

Put another way, VCN occurred in 13.1% of participants with right hemisphere stroke and 5.5% of those with left hemisphere stroke. SCN occurred in 16.3% of participants with right hemisphere stroke and 9.6% of those with left hemisphere stroke. The co-occurrence was less common than either type of neglect in isolation after left hemisphere stroke and less common than SCN alone in right hemisphere stroke. (Figure 4).

### 3.2. Partially Distinct Areas of Infarct Contribute to VCN and SCN

For right hemisphere stroke participants, the lesion load across the right hemisphere vascular territories, along with age, predicted 45.0% of the variance in incidence of left VCN (*p* < 0.00001). The presence of VCN was independently predicted by age (*p* = 0.002) and lesion load in the right ACA territory (*p* = 0.001) and right MCA occipital portion (*p* = 0.03). The lesion load across the right hemisphere vascular territories, along with age, predicted 15.5% of the variance in incidence of left SCN (*p* = 0.0048). None of the lesioned areas alone or age independently predicted left SCN. Refer to Table 2 and Table 3 for odds ratios and 95% confidence intervals.

In the left hemisphere stroke, none of the lesioned areas alone or age independently or together predicted the right VCN. However, the lesion load across the left hemisphere vascular territories, along with age, predicted 49.0% of the variance in the incidence of right SCN (*p* = 0.0063). The absence of any damage to the left PCA temporal region predicted the absence of the right SCN perfectly and was omitted from the model. Right SCN was independently predicted lesion load in the left ACA (*p* = 0.024), left PCA occipital region (*p* = 0.015), and age (*p* = 0.021). Refer to Table 4 and Table 5 for odds ratios and 95% confidence intervals.

### 3.3. The Contribution of Areas of Hypoperfusion (Estimated with FLAIR Vessel Hyperintensities) in Accounting for the Variance in Each Type of Neglect

In the right hemisphere stroke participants, adding the FVH values to the model for left VCN improved the amount of variance explained from 44.5% to 55.0% (*p* < 0.00001). In this model (like the model without FVH), age (*p* = 0.030), lesion load in the right ACA territory (*p* = 0.003), and MCA occipital region (*p* = 0.03) independently predicted left VCN. Likewise, adding the FVH values to the model for the left SCN improved the amount of variance explained from 15.6% to 20.1% (*p* = 0.012). In this model, FVH (indicating hypoperfusion) in the parietal cortex (*p* = 0.043) and age (*p* = 0.009) independently predicted left SCN. Refer to Table 6 and Table 7 for odds ratios and 95% confidence intervals.

In the left hemisphere stroke participants, adding the FVH values to the model for right VCN improved the amount of variance explained from 8.4% (ns) to 20.7% (*p* = 0.03). None of the lesioned areas alone or age independently predicted right VCN (see Table 8). However, adding FVH to the model for right SCN improved the amount of variance explained from 49.1% (*p* = 0.0063) to 100% (incalculable odds ratios for each independent variable, likely due to the relatively small number of participants with neglect due to left hemisphere lesions).

## 4. Discussion

Here, we evaluated three hypotheses. We partially confirmed the first hypothesis, that VCN and SCN dissociate, even on the same tasks/with the same task demands; and VCN is more common after right hemisphere stroke. However, SCN was more common after right than left hemisphere stroke. The co-occurrence was relatively uncommon after stroke in either hemisphere. Results are consistent with most previous studies that have tested both types of neglect in acute and chronic strokes [4,5,6,9]. However, they contrast with the findings of Rorden et al. [11], in which the two types of neglect were highly correlated. One plausible explanation for the contrasting findings is that patients in the Rorden study [11] had larger strokes, including areas important for both VCN and SCN. The slightly greater frequency of SCN alone (without VCN) after right than left hemisphere stroke (9.3% vs. 8.2%) was unexpected but is likely explained by the larger infarct volume (30 vs. 19 cc) and larger volume of hypoperfusion estimated by the number of FVH (1.7 vs. 1; *p* = 0.008) in right versus left hemisphere strokes. Alternatively, the results may simply reflect the distribution of lesion sites in right and left hemisphere stroke participants in this study or the relatively small number of individuals with left hemisphere stroke. Most studies of the incidence of neglect have tested only VCN, which is more common after the right hemisphere stroke in this and other studies. This bias toward evaluating VCN may account for the common assertion that neglect is more common and severe in right than left hemisphere stroke (although we found that both types of neglect are more common after right than left hemisphere stroke).

We also confirmed the second hypothesis, that partially distinct areas of infarct contribute to VCN and SCN. While lesion load in right MCA—occipital and right ACA regions (and age) independently predicted the presence of left VCN, right parietal hypoperfusion (indicated by FVH), and other areas together were necessary for predicting the presence of SCN in the same individuals. Unlike previous studies, we did not carry out voxel-based analyses to identify smaller areas most strongly associated with either type of neglect. Similarly, in left hemisphere stroke, right VCN was only predicted by all of the left hemisphere regions together, along with age and FVH. In contrast, right SCN was predicted by the lesion load in left PCA occipital and left ACA regions (and age). We did confirm that more frontal (ACA) infarcts were more associated with VCN, and more ventral (PCA) infarcts were associated with SCN. We did not evaluate the proposal that the dorsal visual processing stream, when damaged, is more responsible for VCN versus the ventral stream being more responsible for SCN [15].

Finally, we confirmed the last hypothesis, that information about the areas of hypoperfusion (estimated with FLAIR hyperintense vessel) contributes additional information to areas of dysfunction (infarct and/or hypoperfusion) that account for the variance in each type of neglect. The estimated percentage of variance explained by the anatomical model (based on pseudo r^2^) improved from 44.5% to 55.0% for left VCN, from 15.6% to 20.1% for left SCN, from 8.0% to 20.7% for right VCN, and from 49.1% to 100% for right SCN when we added data on the location and number of FHVs in each area. These results demonstrate that brain mapping studies in acute stroke can include approximate areas of hypoperfusion beyond the infarct that may account for acute deficits, even when perfusion MRI is not available.

Limitations of our study include the relatively small number of participants with left hemisphere stroke and the smaller infarct volume and volume of hypoperfusion (estimated with FVH) in left compared to right hemisphere stroke. These two limitations both reflect the fact that individuals with larger left hemisphere strokes often had comprehension deficits that precluded them from understanding the task. But these limitations may have precluded us from identifying the reliable location of infarct and/or hypoperfusion responsible for VCN and SCN after left hemisphere stroke. The pseudo r^2^ of 1 identified for the model of right SCN is clearly not reliable but reflects the small number of people with the right SCN in this study. Another limitation is that we did not have perfusion-weighted images to identify the more exact areas of hypoperfusion that could cause neglect in our participants. But early studies have shown that FVH accurately reflects both the volume [22,24] and location of hypoperfusion, as measured in participants with both FLAIR and perfusion-weighted imaging at the same time, although clearly arterial spin labeling (ASL) MRI or dynamic contrast tracking perfusion-weighted imaging would have been more sensitive for the precise location and quantity of hypoperfusion. Finally, we did not evaluate the contribution of white matter tracts in accounting for each type of neglect. Previous studies have shown that disruption of white matter tracts can account for hemispatial neglect [25,26]. Future studies will further assess the contributions of disrupted white matter tracts and atrophy in each hemisphere.

## 5. Conclusions

Despite its limitations, this study provided confirmatory evidence on the dissociation and relative frequency of VCN and SCN in acute stroke, which should lead to better detection of hemispatial neglect in the left as well as the right hemisphere stroke. We also provided new evidence on the potential contribution of FVH ratings in accounting for both VCN and SCN. Thus, clinical MRI with FLAIR sequences can provide valuable additional information about hypoperfusion that may be causing neglect and should be useful in guiding reperfusion therapies to improve neglect and other cognitive deficits after stroke.

## Figures and Tables

**Table 1:** Demographic information on participants with left and right hemisphere stroke.

	Left Hemisphere Stroke n = 73	Right Hemisphere Stroke n = 160	Difference (*p*-Value by *t*-Test or X^2^)
Age Mean (SD)	61.3 (12.0)	62.0 (14.6)	ns
Education Mean (SD)	14.4 (3.4)	13.4 (3.3)	t = 1.7; df231 *p* = 0.04
Sex F:M (% female)	34:36 * (48.6%)	76:79 * (49.0%)	ns
Race			
Black	40 (54.8%)	82 (51.2%)	ns
White	29 (39.8%)	70 (43.8%)	ns
Asian	1 (1.4%)	1 (0.6%)	ns
Native American	0	0	ns
Other/Not given	3 (4.1%)	7 (4.4%)	ns
Infarct Volume	19.0 (24.7)	31.6 (54.7)	ns
Total FVH (marker of hypoperfusion volume)	1 (0.19)	1.7 (0.16)	t = −2.7; df231 *p* = 0.008

* The remainder are unknown or undeclared gender. ns = non-significant.

**Table 2:** Model for left viewer-centered neglect without FHV.

	Odds Ratio	z	*p*-Value	95% Confidence Interval	
Age	1.09	3.05	0.002	1.03	1.15
Right ACA	1.0002	3.17	0.001	1.00007	1.0003
Right MCA-F	0.999999	−0.03	0.97	0.9999	1.0001
Right MCA-P	1.000006	0.17	0.86	0.9999	1.0001
Right MCA-T	1.000005	0.14	0.89	0.9999	1.0001
Right MCA-O	1.0002	2.15	0.03	1.00002	1.0004
Right PCA-T	1.0005	1.20	0.23	0.9997	1.001
Right PCA-O	1.00002	0.25	0.80	0.9999	1.0002

ACA = anterior cerebral artery; MCA-F = middle cerebral artery—frontal portion; MCA-P = middle cerebral artery—parietal portion; MCA-T = middle cerebral artery—temporal portion; MCA-O = middle cerebral artery—occipital portion; PCA-T = posterior cerebral artery—temporal portion; PCA-O = posterior cerebral artery—occipital portion.

**Table 3:** Model for left stimulus-centered neglect without FHV.

	Odds Ratio	z	*p*-Value	95% Confidence Interval	
Age	1.05	2.50	0.01	1.01	1.09
Right ACA	1.00008	1.56	0.12	0.99998	1.0002
Right MCA-F	1.00002	0.87	0.38	0.99997	1.0001
Right MCA-P	0.99995	−1.28	0.20	0.9999	1.00002
Right MCA-T	1.00003	1.17	0.24	0.99998	1.0001
Right MCA-O	1.0001	0.69	0.49	0.9999	1.0002
Right PCA-T	0.9999	−0.52	0.60	0.999	1.0004
Right PCA-O	1.00005	0.87	0.39	0.9999	1.0002

ACA = anterior cerebral artery; MCA-F = middle cerebral artery—frontal portion; MCA-P = middle cerebral artery—parietal portion; MCA-T = middle cerebral artery—temporal portion; MCA-O = middle cerebral artery—occipital portion; PCA-T = posterior cerebral artery—temporal portion; PCA-O = posterior cerebral artery—occipital portion.

**Table 4:** Model for right viewer-centered neglect without FHV.

	Odds Ratio	z	*p*-Value	95% Confidence Interval	
Age	1.04	0.90	0.37	0.95	1.14
Left ACA	1.00004	0.26	0.80	0.9998	1.0003
Left MCA-F	0.99995	−0.50	0.62	0.9998	1.0001
Left MCA-P	1.00004	0.64	0.53	0.9999	1.0002
Left MCA-T	0.9999	−0.61	0.54	0.9995	1.0002
Left MCA-O	1.0003	0.73	0.46	0.9996	1.001
Left MCA-I	1.0002	0.25	0.81	0.999	1.002
Left PCA-T	0.99995	−0.05	0.96	0.998	1.002
Left PCA-O	1.00005	0.57	0.57	0.9999	1.0002

ACA = anterior cerebral artery; MCA-F = middle cerebral artery—frontal portion; MCA-P = middle cerebral artery—parietal portion; MCA-T = middle cerebral artery—temporal portion; MCA-O = middle cerebral artery—occipital portion; PCA-T = posterior cerebral artery—temporal portion; PCA-O = posterior cerebral artery—occipital portion.

**Table 5:** Model for right stimulus-centered neglect without FHV.

	Odds Ratio	z	*p*-Value	95% Confidence Interval	
Age	1.17	2.31	0.021	1.03	1.35
Left ACA	1.0002	2.26	0.024	1.00003	1.0004
Left MCA-F	0.99996	−0.53	0.60	0.9998	1.0001
Left MCA-P	1.00004	0.48	0.63	0.9999	1.0002
Left MCA-T	0.9997	−0.33	0.75	0.998	1.002
Left MCA-O	0.999	−0.95	0.34	0.997	1.001
Left MCA-I	0.99997	−0.04	0.97	0.998	1.002
Left PCA-O	1.001	2.42	0.015	1.0003	1.002

ACA = anterior cerebral artery; MCA-F = middle cerebral artery—frontal portion; MCA-P = middle cerebral artery—parietal portion; MCA-T = middle cerebral artery—temporal portion; MCA-O = middle cerebral artery—occipital portion; PCA-T = posterior cerebral artery—temporal portion; PCA-O = posterior cerebral artery—occipital portion.

**Table 6:** Model for left viewer-centered neglect with FHV.

	Odds Ratio	z	*p*-Value	95% Confidence Interval	
Frontal FHV	0.84	−0.20	0.84	0.14	4.88
Temporal FHV	1.69	0.94	0.35	0.56	5.10
Parietal FHV	0.22	−1.81	0.07	0.04	1.13
Insular FHV	1.83	0.92	0.36	0.51	6.62
Age	1.07	2.11	0.030	1.005	1.14
Right ACA	1.0003	2.93	0.003	1.0001	1.0004
Right MCA-F	1.00001	0.22	0.83	0.9999	1.0001
Right MCA-P	1.00003	0.65	0.51	0.9999	1.0001
Right MCA-T	1.00004	0.97	0.33	0.99996	1.0001
Right MCA-O	1.0003	2.13	0.03	1.00002	1.0006
Right PCA-T	1.004	1.19	0.23	0.998	1.01
Right PCA-O	0.9999	−0.80	0.43	0.9996	1.0002

ACA = anterior cerebral artery; FHVs = FLAIR hyperintense vessels; PCA = posterior cerebral artery; MCA-F = middle cerebral artery—frontal portion; MCA-P = middle cerebral artery—parietal portion; MCA-T = middle cerebral artery—temporal portion; MCA-O = middle cerebral artery—occipital portion; PCA-T = posterior cerebral artery—temporal portion; PCA-O = posterior cerebral artery—occipital portion.

**Table 7:** Model for left stimulus-centered neglect with FHV.

	Odds Ratio	z	*p*-Value	95% Confidence Interval	
ACA FHV	0.53	−0.81	0.42	0.11	2.49
PCA FHV	0.97	−0.04	0.96	0.23	4.06
Frontal FHV	0.73	−0.60	0.55	0.27	2.01
Temporal FHV	0.69	−0.95	0.34	0.32	1.49
Parietal FHV	2.31	2.02	0.043	1.02	5.19
Insular FHV	1.31	0.62	0.54	0.56	3.04
Age	1.06	2.63	0.009	1.01	1.10
Right ACA	1.0001	1.95	0.05	0.999999	1.0002
Right MCA-F	1.00003	0.99	0.32	0.99997	1.0001
Right MCA-P	0.9999	−1.98	0.05	0.9999	0.999999
Right MCA-T	1.00004	1.19	0.23	0.99998	1.0001
Right MCA-O	1.0001	0.86	0.39	0.9999	1.0003
Right PCA-T	0.9998	−0.56	0.57	0.999	1.0004
Right PCA-O	1.0001	1.02	0.31	0.9999	1.0002

ACA = anterior cerebral artery; FHVs = FLAIR hyperintense vessels; PCA = posterior cerebral artery; MCA-F = middle cerebral artery—frontal portion; MCA-P = middle cerebral artery—parietal portion; MCA-T = middle cerebral artery—temporal portion; MCA-O = middle cerebral artery—occipital portion; PCA-T = posterior cerebral artery—temporal portion; PCA-O = posterior cerebral artery—occipital portion.

**Table 8:** Model for right viewer-centered neglect with FHV.

	Odds Ratio	z	*p*-Value	95% Confidence Interval	
Frontal FHV	0.59	−0.83	0.41	0.17	2.07
Temporal FHV	2.04	1.72	0.09	0.91	4.59
Parietal FHV	0.53	−1.05	0.29	0.17	1.72
Insular FHV	1.49	0.83	0.41	0.58	3.87
Age	1.04	1.83	0.07	1.00	1.08
Left ACA	1.001	1.51	0.13	0.9997	1.003
Left MCA-F	1.003	0.34	0.74	0.98	1.02
Left MCA-P	0.98	−0.88	0.38	0.92	1.03
Left MCA-T	0.97	−0.48	0.63	0.88	1.08
Left MCA-O	1.16	0.62	0.54	0.72	1.88
Left MCA-I	0.09	−0.30	0.77	1.12 × 10^−8^	740,296.7
Left PCA-T	1.004	0.32	0.75	0.98	1.03
Left PCA-O	1.0008	0.65	0.52	0.998	1.003

ACA = anterior cerebral artery; FHVs = FLAIR hyperintense vessels; PCA = posterior cerebral artery; MCA-F = middle cerebral artery—frontal portion; MCA-P = middle cerebral artery—parietal portion; MCA-T = middle cerebral artery—temporal portion; MCA-O = middle cerebral artery—occipital portion; MCA-I = middle cerebral artery—insular portion; PCA-T = posterior cerebral artery—temporal portion; PCA-O = posterior cerebral artery—occipital portion.

## Data Availability

The raw data supporting the conclusions of this article will be made available by the authors upon request.
